# Umbilical Vein Catheter Protruding Through a Pulmonary Vein in a Patient with an Infracardiac Type Total Abnormal Pulmonary Venous Drainage

**DOI:** 10.1007/s00246-019-02094-3

**Published:** 2019-03-30

**Authors:** Luc Bruyndonckx, Lucia J. M. Kroft, Vincent Bekker, Arno A. W. Roest, Roel L. F. van der Palen

**Affiliations:** 1 Department of Paediatric Cardiology, Leiden University Medical Center, Albinusdreef 2, 2333 ZA Leiden, The Netherlands; 2 Translational Research in Immunology and Inflammation, University of Antwerp, Universiteitsplein 1, 2610 Antwerp, Belgium; 3 Department of Radiology, Leiden University Medical Center, Albinusdreef 2, 2333 ZA Leiden, The Netherlands; 4 Department of Neonatology, Leiden University Medical Center, Albinusdreef 2, 2333 ZA Leiden, The Netherlands

**Fig. 1:**
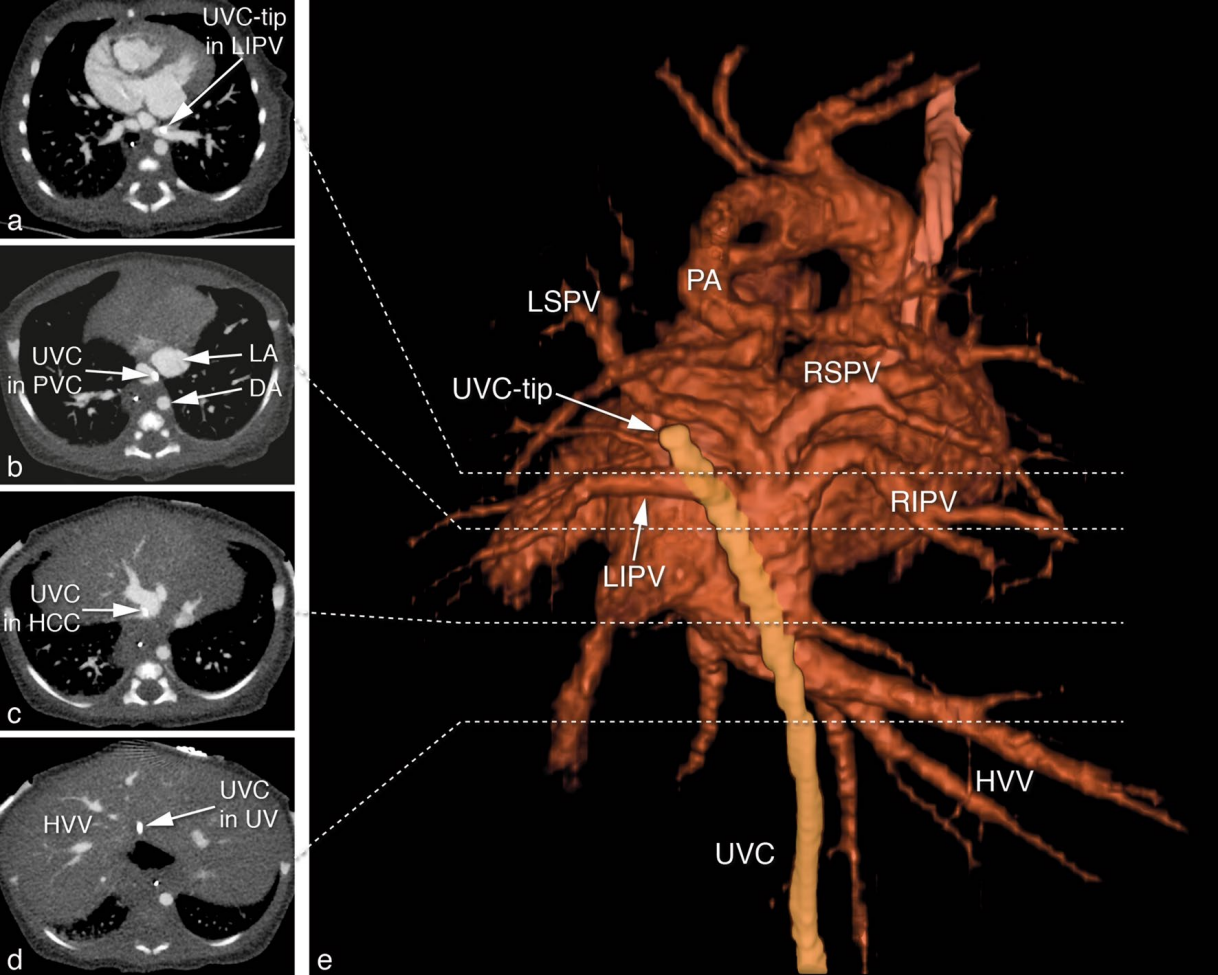
CT images demonstrating the presence of the UVC in a pulmonary vein

**Fig. 2:**
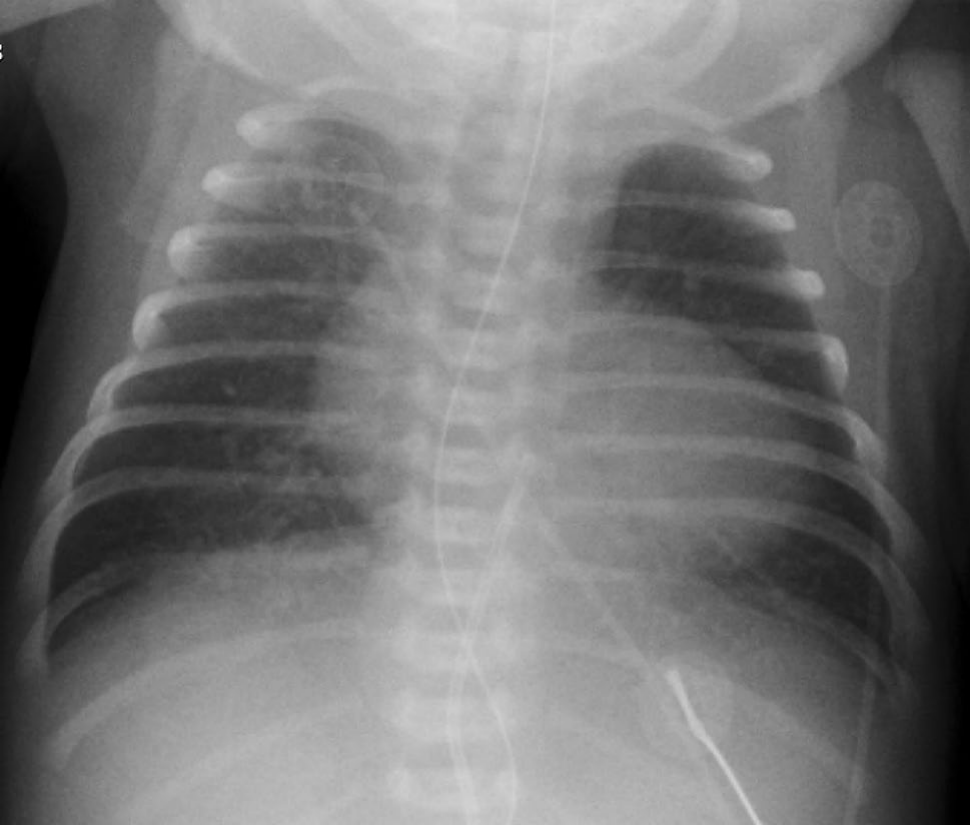
Chest X-ray, suggesting the UVC was positioned rather deep and to the left

A girl born at a gestational age of 36 weeks presented with cyanosis. Echocardiography led to the diagnosis of right isomerism, a complete atrioventricular septal defect, pulmonary atresia, an aortic valve arising from the right ventricle and a total abnormal pulmonary venous drainage (TAPVD) of the infracardiac type, with a collector vein connecting to a liver vein, draining into the left-sided atrium. After venous umbilical catheter placement, alprostadil was started, whereafter saturation increased to 85–90%. Computed tomography (CT) (Fig. 1), performed to further investigate pulmonary venous return, demonstrated the presence of the umbilical vein catheter (UVC) tip in the left inferior pulmonary vein (LIPV) (a), passing the TAPVD collector (b, PVC = pulmonary vein collector, LA = left atrium, DA = descending aorta), the hepatocaval confluence (HCC) (c) and the umbilical vein (UV) (d, HVV: hepatic veins) shown in a 3D surface rendering representation from posterior, with the aorta removed (e, PA = pulmonary artery, RIPV: Right inferior pulmonary vein, RSPV = right superior pulmonary vein, LIPV = Left inferior pulmonary vein), with the umbilical catheter in yellow. Based on the CT, the umbilical venous catheter was removed. In retrospect, the umbilical vein catheter was positioned rather deep and to the left that may have suspected malposition on the chest X-ray (Fig. 2) that was performed prior to the CT scan.

Awareness of the possibility of an abnormal position of the umbilical venous catheter in the presence of an infracardiac TAPVD is warranted since catheter-related endothelial damage and thrombus formation could lead to stenosis of the pulmonary collector vein prompting urgent intervention.

